# Integrated analysis of single cell‐RNA sequencing and Mendelian randomization identifies lactate dehydrogenase B as a target of melatonin in ischemic stroke

**DOI:** 10.1111/cns.14741

**Published:** 2024-05-03

**Authors:** Fei Shi, Guiyun Zhang, Jinshi Li, Liang Shu, Cong Yu, Dabin Ren, Yisong Zhang, Ping Zheng

**Affiliations:** ^1^ Department of Neurovascular Intervention and Neurosurgery, Shanghai General Hospital Shanghai Jiaotong University, School of Medicine Shanghai China; ^2^ Department of Neurology Shanghai Pudong New area People's Hospital Shanghai China; ^3^ Department of Neurology Shanghai Ninth People's Hospital Shanghai China; ^4^ Department of Neurosurgery Shanghai Pudong New area People's Hospital Shanghai China

**Keywords:** causal effect, ischemic stroke, LDHB, Mendelian randomization

## Abstract

**Aims:**

Despite the success of single‐cell RNA sequencing in identifying cellular heterogeneity in ischemic stroke, clarifying the mechanisms underlying these associations of differently expressed genes remains challenging. Several studies that integrate gene expression and gene expression quantitative trait loci (eQTLs) with genome wide‐association study (GWAS) data to determine their causal role have been proposed.

**Methods:**

Here, we combined Mendelian randomization (MR) framework and single cell (sc) RNA sequencing to study how differently expressed genes (DEGs) mediating the effect of gene expression on ischemic stroke. The hub gene was further validated in the in vitro model.

**Results:**

We identified 2339 DEGs in 10 cell clusters. Among these DEGs, 58 genes were associated with the risk of ischemic stroke. After external validation with eQTL dataset, lactate dehydrogenase B (LDHB) is identified to be positively associated with ischemic stroke. The expression of LDHB has also been validated in sc RNA‐seq with dominant expression in microglia and astrocytes, and melatonin is able to reduce the LDHB expression and activity in vitro ischemic models.

**Conclusion:**

Our study identifies LDHB as a novel biomarker for ischemic stroke via combining the sc RNA‐seq and MR analysis.

## Graphical abstract

LDHB is increased in ischemic stroke and is consistent with NIHSS score in human patients. LDHB is identified as a novel biomarker for ischemic stroke via combining the sc RNA‐seq and MR analysis.

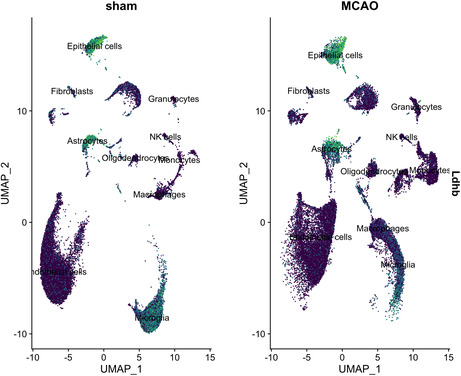

## 1 INTRODUCTION

Stroke is a prevalent neurological disease, with ischemic stroke (IS) accounting for approximately 70%–80% of all adult stroke cases, and its incidence rate has been steadily rising each year.^1^ The average stroke incidence is 120–180 per 100,000 people annually, with a higher occurrence among men. According to the Global Burden of Disease Study, which provides estimates for 2019, the global age‐standardized incidence rate of ischemic stroke was 132 per 100,000 person‐years. For patients aged more than 65 years, the incidence rate increases to 2875/100,000 in men and 1839/100,000 in women per year.^2^ The conventional treatments for IS consist of mechanical thrombectomy and intravenous thrombolysis.^3^, ^4^ In contrast, the emerging treatment approaches encompass cellular therapy and noninvasive brain stimulation.^5^ Nevertheless, the effectiveness of thrombolysis interventions is limited by the narrow time window, typically within 4.5 h, and sometimes extended up to 6 h,^6^ therefore, to predict the occurrence of ischemic stroke is very critical in clinical session.

Single cell RNA sequencing is an advanced sequencing technique to detect the cellular heterogeneity.^7^ Previous studies have applied this technique in ischemic stroke to show that differently expressed genes (DEGs) in different cell types and identified the stroke‐specific cell cluster. However, the exact role of these DEGs has not been well linked to the initiation of an ischemic stroke. The causal effects of cellular DEGs on IS have not been firmly established. To address this, Mendelian randomization (MR) emerges as a valuable tool to estimate the causal effect of an exposure on disease onset and prognosis.^8^ The MR method utilizes genetic variants correlated with risk factors as instrumental variables (IV). This effectively overcomes the challenges of confounding and reverse causality.^8^

However, in the context of DEG analysis, identifying genetic variants solely associated with a single component can be challenging. To address this issue, we employed gene expression quantitative trait loci (eQTL) as the exposure variable and eQTL allow us to estimate the direct effects of each component of the gene profile on IS while additionally adjusting for pleiotropy in instrumental variables.^9^ We further validated the results in oxygen‐glycose depression model. Thus, our study integrated scRNA‐seq and MR analyses to test the hypothesis that differentially expressed genes might be causally associated with the risk of IS. By doing so, we aimed to shed light on the potential causal relationships between cellular DEGs and the development of IS.

## 2 RESULTS

**FIGURE 1:**
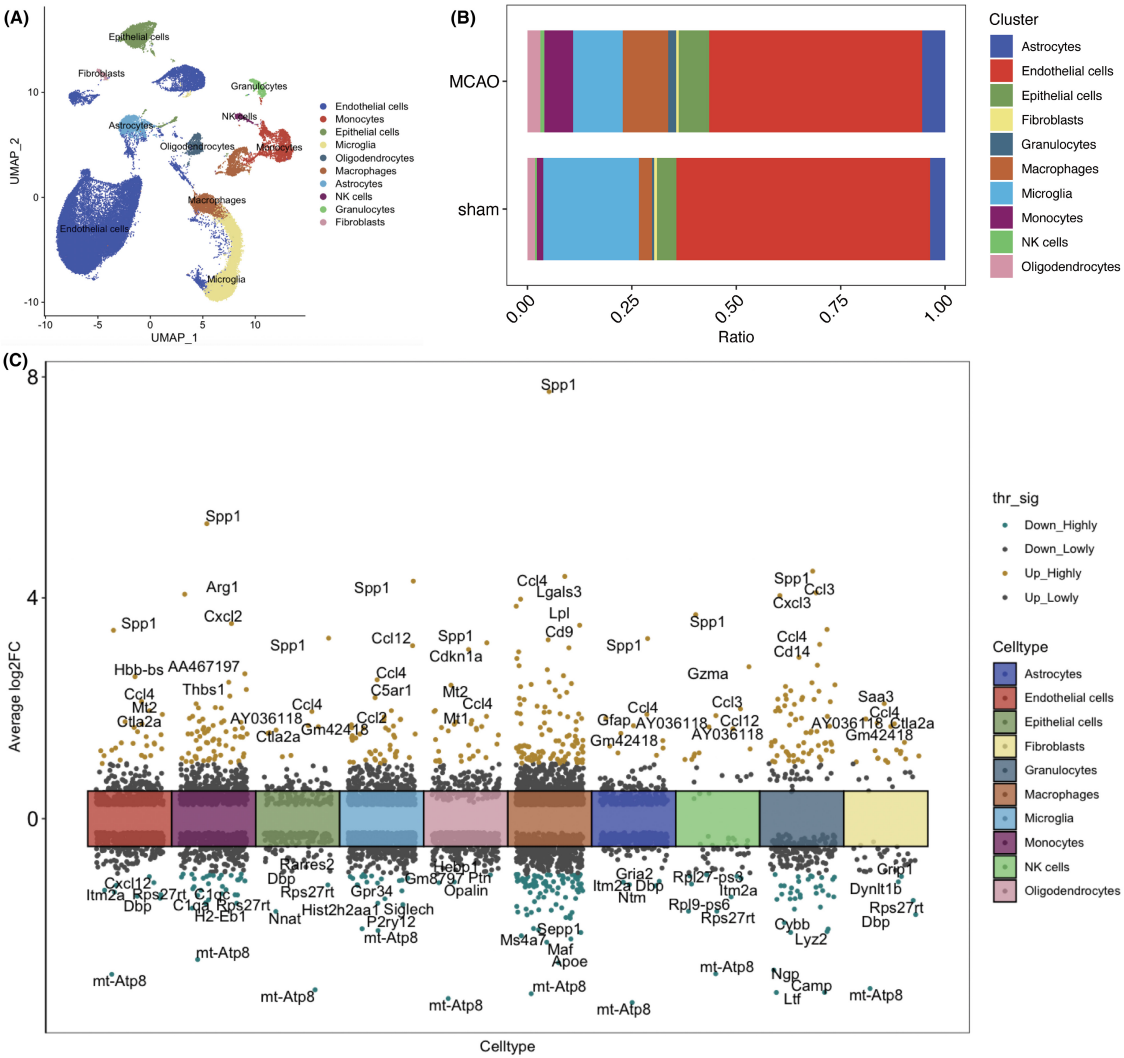
The single‐cell RNA‐sequencing results for ischemic stroke mice. (A) Umap shows the 10 cell clusters. (B) The bar plot shows the cell ratio between ischemic stroke mice and sham mice. (C) The top 5 upregulated and downregulated DEGs in each cluster.

To confirm the cellular heterogeneity of ischemic stroke, we analyzed the sc‐RNA seq data from GSE 174574, which had three IS and three sham mice as there was no human stroke sc‐RNA seq data. The detailed information regarding the samples were reported previously.^10^ We first showed the cell clusters in UMAP (Figure 1A). We then showed the cell ratio between the two groups, and we found 10 cell clusters: astrocytes, endothelial cells, epithelial cells, fibroblasts, granulocytes, macrophages, microglia, monocytes, NK cells, and oligodendrocytes (Figure 1B). We next identified 2339 DEGs (log_2_FC >0.5) in the brains from sham and MCAO mice in 10 cell groups, and top 5 upregulated or downregulated genes were listed (Figure 1C).

**FIGURE 2:**
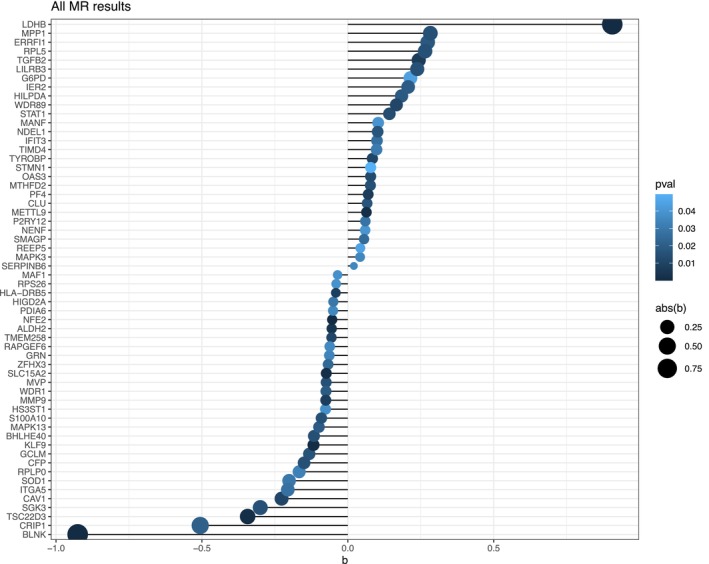
The lollipop plot shows all MR results of each DEG. The *X*‐axis is *R* coefficient of correlation. The *Y*‐axis is each DEG in an ischemic stroke. The color scale indicates the p value.

To further investigate the role of these DEGs, we extracted the eqtl (genetic variation) of these 2339 DEGs from GSWA summary website as exposure variables and the ischemic stroke (ebi‐a‐GCST005843) as the outcome variable for the two‐sample MR analysis. We found 28 DEGs were positively associated with the outcome, and 30 DEGs were negatively correlated with the outcome (Figure 2).

**FIGURE 3:**
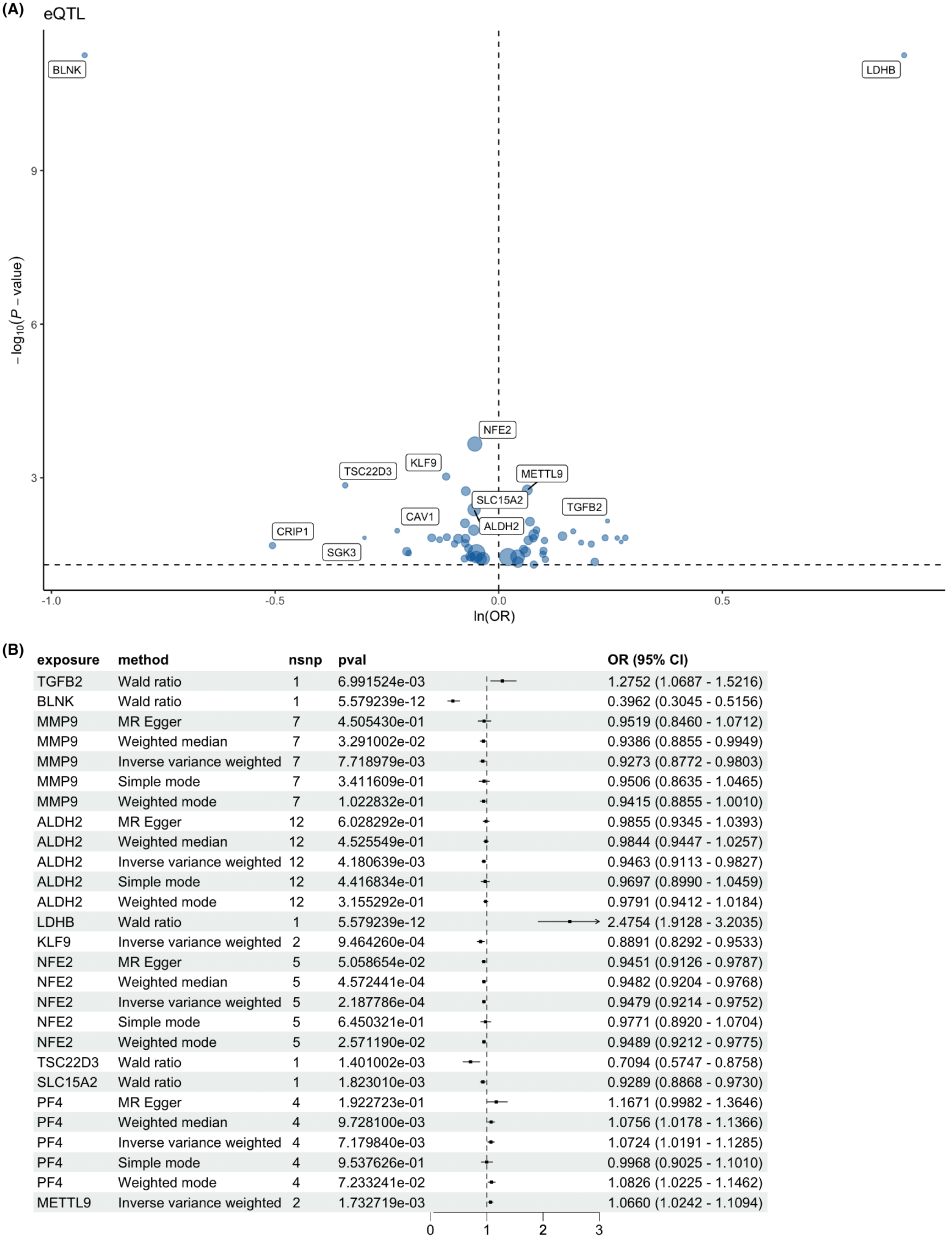
The MR results of LDHB on ischemic stroke. (A) The bubble plot of hub gene (eQTL) on ischemic stroke. (B) The forest plot of hub gene (eQTL) in an ischemic stroke.

Among the 28 DEGs, lactate dehydrogenase B (LDHB) was found to be a top risk factor in ischemic stroke. However, its role in ischemic stroke has not been fully investigated. Given the best causal estimation, elevated levels of LDHB were significantly associated with an increased risk of IS (OR [95% CI] = 2.4754 [1.9128–3.2035], *p* = 5.579e−12, Figure 3).

**FIGURE 4:**
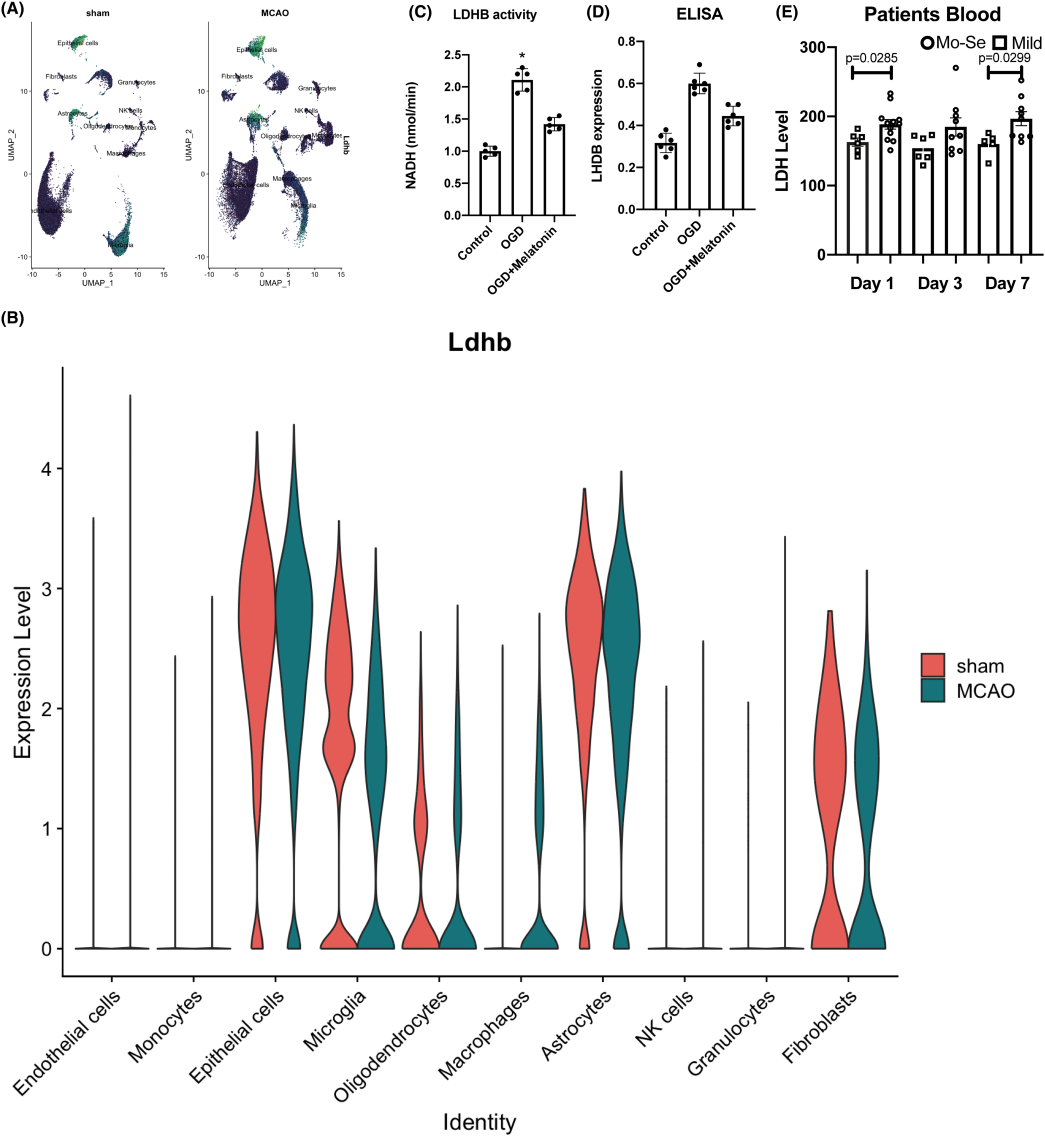
The distribution of Ldhb in sc RNA‐seq data. (A, B) The cellular distribution of Ldhb between sham and MCAO mice. (C) The LDHB activity in OGD models. (D) The LDHB expression in OGD models. (E) LDH level in patients' blood.

To validate the expression of LDHB in stroke, we found it mainly distributed in microglia, astrocytes, and macrophage (Figure 4A,B). To confirm the role of LDHB in ischemic stroke, we adopted the OGD model to assess the expression and activity of LDHB. We found both the expression and activity of LDHB increased in the macrophages treated with OGD, and melatonin treatment could partly reduce them (Figure 4C,D). LDH level is further validated in ischemic stroke patients' blood. In Day one and Day seven group, the LDH level is increased in higher NIHSS group (NIHSS >5) compared to lower NIHSS group (NIHSS <5).

**FIGURE 5:**
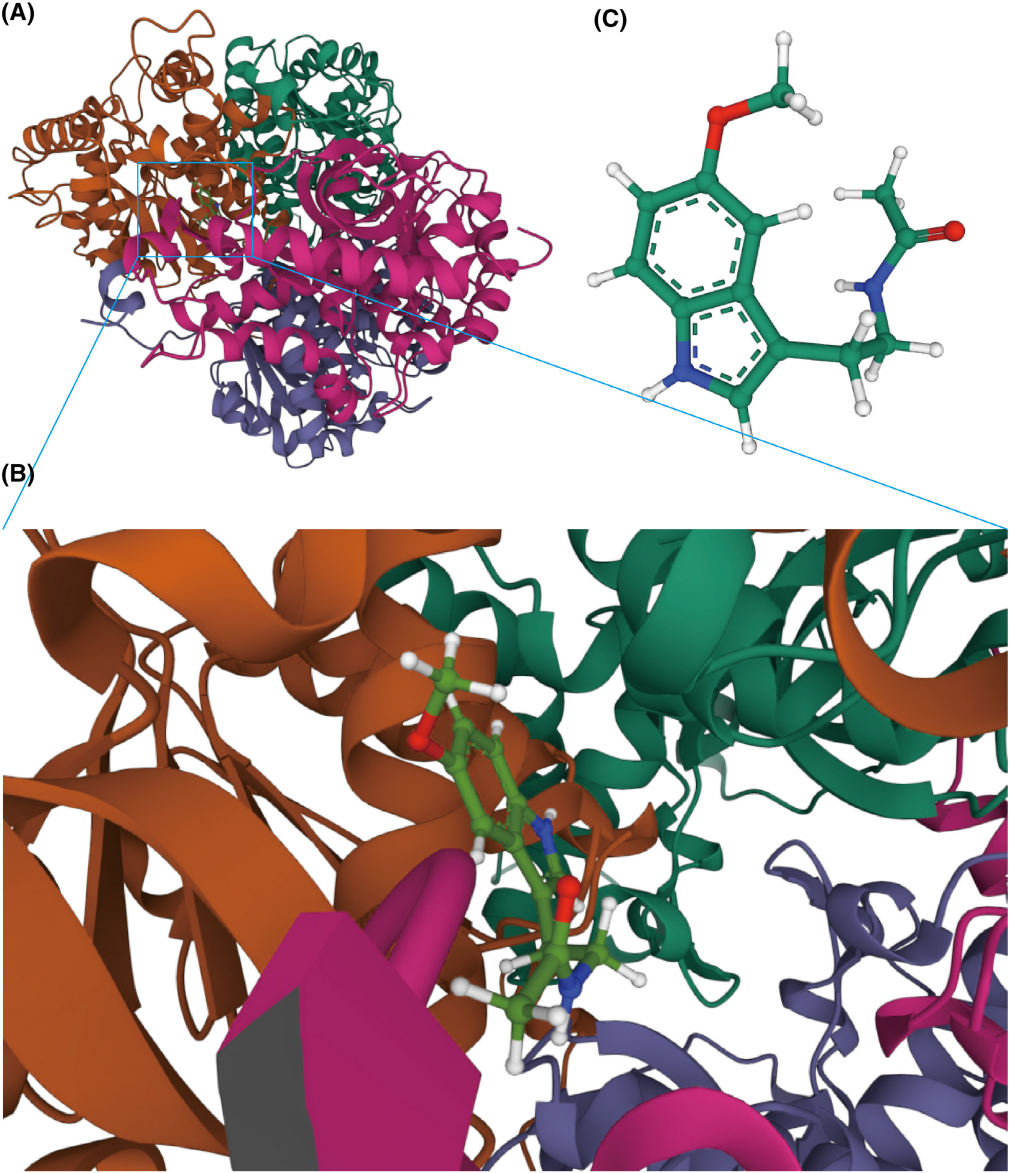
Binding mode of melatonin to LDHB by molecular docking. (A) Enlarged three‐dimensional structures of the binding pockets were shown by AutoDock software. (B) 3D interactions of melatonin and LDHB. (C) Cartoon representations overlay of the crystal structures of melatonin.

As melatonin was previously shown to affect the expression of LDHB,^11^, ^12^ we further investigated the affinity of the melatonin for its target: LDHB with molecular docking analysis. The binding poses and interactions were obtained with Autodock Vina v.1.2.2, and binding energy for each interaction was generated (Figure 5A–C). Results showed that melatonin is bound to LDHB through visible hydrogen bonds and strong electrostatic interactions. Moreover, the hydrophobic pockets of each target were occupied successfully. Tubulin alpha chain and tubulin beta chain had low binding energy of −7.182 and −7.148 kcal/mol, indicating highly stable binding.

## 3 DISCUSSION

Currently, there are few studies combing the sc RNA‐seq and Mendelian randomization, where we applied this approach to estimate the causal association between hub genes and the risk of IS. Combining MR with scRNA‐seq can provide valuable insights into causal relationships between genetic variants, gene expression, and complex traits, which is useful in comparing expression levels across cell types, examining expression patterns within specific cell populations, or evaluating the impact of genetic variants on gene expression heterogeneity. Our study demonstrated that LDHB had a positive causal effect on the risk of IS, which showed significantly causal associations with vessel infarction.

In the sc RNA‐seq analysis, our findings were robust and consistent with previous reports, which shows the post‐stroke immune cell heterogeneity.^13^ Among the 2339 DEGs, we applied the MR analysis and identified 58 genes that might be involved in the occurrence of ischemic stroke.

Our single cell results and other studies showed the LDHB expression is increased in the ischemic stroke, while it is especially expressed in macrophage. LDHB is an enzyme involved in the conversion of lactate to pyruvate in the glycolytic pathway.^14^, ^15^ In the context of ischemic stroke, LDHB and lactate metabolism play a role in the response to the ischemic injury, and high LDH level indicates poor neurological outcome.^16^ During ischemia, there is a reduction in oxygen and glucose supply to the affected area of the brain, which leads to a switch in cellular metabolism from aerobic metabolism (using oxygen) to anaerobic metabolism (without oxygen), resulting in an increase in lactate production. LDHB is responsible for the conversion of lactate to pyruvate. The presence of LDHB allows the cell to regenerate NAD+, which is necessary for glycolysis to continue. Glycolysis is an essential pathway for energy production, and it becomes a primary source of adenosine triphosphate (ATP) during ischemic conditions.^17^ Research suggests that LDHB, and the regulation of lactate metabolism may have implications for the severity of ischemic stroke and the subsequent damage to brain tissue. One report showed that LDH might be a risk predictor of poor prognosis and all‐cause death of acute ischemic stroke, and the mortality rates at 3‐month, 6‐month, 12‐month, and 18‐month were 2.46%, 3.69%, 4.92%, and 5.33%, respectively.^16^ It can also predict unfavorable outcomes after rt‐PA thrombolysis is IS patients.^18^ Regarding the clinical translation, removal of LDHB leads to an increase in symmetric neuronal terminal division.^19^ Kang et al. found that knockout of pyruvate kinase M2 (PKM2) could reduce the expression of LDHB level after global cerebral ischemia,^20^ which explained why LDHB increased in ischemic stroke.

Our study acknowledges several limitations. First, we cannot entirely rule out the possibility of horizontal pleiotropy, which could introduce bias in our causal effect estimates.^21^ To address this concern, we employed MR‐PRESSO and MR‐Egger approaches to assess the impact of heterogeneity and pleiotropy on the risk estimates derived from the MR analyses. Fortunately, these assessments indicated that neither heterogeneity nor pleiotropy significantly influenced the observed causal effect. Second, the concept of canalization, where the causal effect of SNP exposure on SNP‐outcome during development is altered by compensatory developmental processes,^22^ may potentially dampen or buffer the association between genetically predicted risk factors and IS. And future studies are needed to investigate whether risk factors can mediate the hub genes and the onset of ischemic stroke. Lastly, we acknowledge that environmental exposures and their interactions with predisposing genetic factors play pivotal roles in the pathogenesis of IS.^23^ Further study applied the MR approach to assess the environmental exposure and its effect for IS, providing a valuable tool to guide clinical studies in the future.

## 4 CONCLUSIONS

Our combined analyses provide genetic evidence to support a positive causal association between high LDHB levels and the increased risk of IS, while melatonin can reduce the LDHB activity in vitro. This result indicates that melatonin targeted LDHB might be a novel target for the primary prevention of IS, especially for the treatment outcome.

## 5 METHODS

### 5.1 Medical ethics

In this study, no animal experiments were involved.

### 5.2 Single‐cell‐sequencing data obtained and processing

The single‐cell data for mouse ischemic stroke model were downloaded from NCBI website:GEO174574. Next, we carry out data quality control. We captured cells with less than 10 percent mitochondrial genes, with a total number of genes ranging from 200 to 10,000 that were expressed in at least three cells. The number of highly variable genes was set at 2000. The six samples were integrated through SCT correction. Then, uMAP method was used to reduce the dimension of data. We used FindAllMarkers to investigate the DEGs in different cell clusters.

### 5.3 MR study design

We applied the two‐sample MR to evaluate the causal effects of gene profiles on ischemic stroke using genetic variables of DEGs from single‐cell RNA sequencing data as IVs according to the previous studies.^24^ We extracted single nucleotide polymorphisms (SNPs) serving as instrumental variables (IVs) for each trait from previously published GWAS data.^25^, ^26^, ^27^ These SNPs were then clumped together to obtain independent genetic variants, ensuring a linkage disequilibrium (LD) *r* ^2^ < 0.001 and a clumping distance of 1000 kb. For the selection of strong IVs, we considered a value of *F*‐statistic greater than ten, which was deemed sufficient to predict the exposures of interest.^28^, ^29^ The study protocols were approved by the ethics committee of the local hospital.

### 5.4 Data for exposure

To enhance the robustness of our analysis and minimize selection bias, we utilized datasets from ethnic groups of European ancestry. The study protocols were ethically approved by the local hospital's ethics committee. Regarding the data for exposure, we obtained SNPs associated with differentially expressed gene (DEG) expression at genome‐wide significance (*p* < 5 × 10^−6^) from a published GWAS study. Summary statistics for cis‐eQTLs stem from the eQTLGen Consortium meta‐analysis of 19,942 transcripts in 31,684 individuals.^30^

### 5.5 Data for outcome

GWAS summary data on IS from six contributing studies (The Nord‐Trøndelag Health Study, Michigan Genomics Initiative, deCODE, UK Biobank, DiscovEHR Collaboration Cohort, and AFGen Consortium) were used including 440,328 participants of European ancestry, which contained 8,296,492 SNPs. The coefficient of each SNP was transformed to log odds ratio (OR) of IS. We extracted GWAS summary data on small vessel infarction and large artery infarction in 198,048 participants of European ancestry performed by the MEGASTROKE consortium and 150,765 stroke patients with large artery infarction. We also included 22,593 participants with cognitive function.

### 5.6 Validation with external datasets

The Mendelian randomization method is used to validate the causal associations between genetic profile and IS. The multivariable MR was performed using five approaches to evaluate the direct causal effect of eQTL on IS.

### 5.7 Molecular docking

To analyze the binding affinities and modes of interaction between the drug candidate and their targets, AutodockVina 1.2.2, a silico protein–ligand docking software was employed.^31^ The molecular structures of LDHB were retrieved from PubChem Compound (https://pubchem.ncbi.nlm.nih.gov/).^32^ The 3D coordinates of Stathmin (PDB ID, 7DBK; resolution, 2.5 Å) were downloaded from the PDB (http://www.rcsb.org/). To conduct the docking analysis, the protein and molecular files underwent conversion into PDBQT format, excluding all water molecules and adding polar hydrogen atoms. The grid box was then centered to encompass the domain of each protein and allow sufficient space for molecular movement. The dimensions of the grid box were set at 30 Å × 30 Å × 30 Å, with a grid point distance of 0.05 nm. The molecular docking studies were carried out using Autodock Vina 1.2.2 (http://autodock.scripps.edu/).

### 5.8 Macrophage culture and OGD model

All culture reagents were bought from ThermoFisher Scientific (USA). The in vitro methods were previously described.^33^ Rat macrophage NR8383 cells were seeded at a density of 106 in a 6‐well plate containing 2 mL of medium per well, and when the cells grew to 50% as previously reported.^34^ For oxygen–glucose deprivation (OGD), the macrophage culture at 14 days in vitro (DIV) was transferred to glucose‐free DMEM medium. The macrophage culture was then placed into an anaerobic incubator (Thermo Scientific 1029) that had been previously initiated, resulting in oxygen levels below 0.1%, and the temperature was maintained at 37°C with 5% CO_2_. After 90 minutes of OGD treatment, the culture was removed from the incubator and immediately fixed for subsequent experiments.

### 5.9 LDHB expression and activity test

For Elisa test of LDHB, a 384‐well SimpleStep ELISA microplate (ab203359) is applied. Human LDHB ELISA kit (ab 183367) is used to measure LDHB protein expression in culture medium. The activity of LDHB is tested by microplate assay ab 140361, which can determine the activity in production of NADH catalyzed by this enzyme. Patients' LDHB level is determined by Radio‐immuno assay in patients' blood. The peripheral blood is collected on Day 1, Day 3, and Day 7 after the ischemic stroke onset.

### 5.10 Statistical analysis

We employed a two‐sample Mendelian randomization (MR) method to assess the causal effects of hub genes on the incidence of ischemic stroke, presenting the results as odds ratios (ORs) with corresponding 95% confidence intervals (CIs). To ensure reliable harmonization of SNP‐exposure and SNP‐outcome, we followed previously described procedures.^35^ To address potential heterogeneity and horizontal pleiotropy across the causal estimates, we implemented five MR approaches: random‐effect inverse‐variance weighted (IVW), weighted median, MR‐Egger, simple mode, and weighted mode. The IVW regression approach assumes that either all genetic variants are valid instruments or that there is no evidence of pleiotropy effect.^36^ Meanwhile, the weighted median analysis was utilized to assess robustness in consistent estimation if more than 50% of the instrumental variables are valid.^37^ To investigate potential horizontal pleiotropy, we conducted MR‐Egger analysis, employing weighted linear regression between SNP‐exposure and SNP‐outcome, and detected its presence by examining the intercept of the MR‐Egger coefficient.^38^ Additionally, the MR‐PRESSO test was used to detect and calibrate potential pleiotropic outliers in the summary‐level MR analyses.^39^ To evaluate heterogeneity, scatter plots and the Cochran Q tests were performed between causal estimates from multiple genetic variants.^40^

In sensitivity analyses, we compared the causal estimates from various MR methods, such as MR‐Egger, penalized weighted median, simple mode, IVW, and weighted mode, to enhance the robustness of our findings. Forest plots were utilized to assess the causal effects of individual SNPs and to further compare them against the causal estimates derived from the IVW and MR‐Egger approaches, which employed all enrolled SNPs. Furthermore, we examined possible directional pleiotropy by observing asymmetry in funnel plots to gauge the reliability of the current MR analyses.

Similarly, we explored the causal estimates between selected eQTLs and ischemic infarction to confirm the causal associations between gene profiles and infarction types. A two‐sided P‐value less than 0.05 was considered statistically significant for a causal association. The comparisons among groups were performed with Mann–Whitney test. All statistical analyses were performed using R software version 3.6.2, utilizing the ‘TwoSampleMR’ package version 0.5.2.^41^

## AUTHOR CONTRIBUTIONS

FS, GYZ, and PZ did the bioinformatical study and MR analysis, CY, JSL, DBR, and SL did the in vitro study and all authors contributed to the draft.

## FUNDING INFORMATION

The work was supported by grants from the Cross Research Fund of Medicine and Engineering of Shanghai Jiaotong University (YG2021QN90). Shanghai Pudong New Area Health Commission.

## CONFLICT OF INTEREST STATEMENT

All authors report they do not have conflict of interest.

## CONSENT FOR PUBLICATION

All authors agreed to submit the paper.

## Data Availability

The data that support the findings of this study are openly available in IEU at https://gwas.mrcieu.ac.uk/datasets/?gwas_id__icontains=&year__iexact=&trait__icontains=ischemic+stroke&consortium__icontains=.
